# Association of Leisure-Time Physical Activity to Cardiovascular Disease Prevalence in Relation to Smoking among Adult Nevadans

**DOI:** 10.1371/journal.pone.0128424

**Published:** 2015-05-26

**Authors:** Masaru Teramoto, Sheniz Moonie, Chad L. Cross, Michelle Chino, Patricia T. Alpert

**Affiliations:** 1 Division of Physical Medicine and Rehabilitation, University of Utah, Salt Lake City, UT, United States of America; 2 School of Community Health Sciences, University of Nevada, Las Vegas, Las Vegas, NV, United States of America; 3 Department of Physical & Life Sciences, Nevada State College, Henderson, NV, United States of America; 4 School of Nursing, University of Nevada, Las Vegas, Las Vegas, NV, United States of America; Hamamatsu University School of Medicine, JAPAN

## Abstract

It is well known that cigarette smoking and physical activity have significant impacts on cardiovascular disease (CVD) mortality and morbidity. Meanwhile, it is of interest to understand whether physical activity protects against CVD for smokers in a similar manner as it does for non-smokers. The present study examined how leisure-time physical activity (LTPA) is associated with the prevalence of CVD in relation to smoking status among adult Nevadans, using data from the 2010 Nevada Behavioral Risk Factor Surveillance System. Of the 3,913 survey respondents, 8.5% self-reported that they had ever been diagnosed with CVD. People with a history of CVD were significantly less likely to engage in LTPA than those with no history of CVD (*p* < 0.05). After adjusting for common sociodemographic variables, it was revealed that people with CVD were twice more likely to not engage in LTPA than their counterparts independent of smoking status. Without taking LTPA into account, the odds of having CVD for current and former smokers was 1.87–2.25 times higher than the odds for non-smokers. Interestingly, however, if LTPA was accounted for, there was no significant difference in the odds of having CVD between current and non-smokers. These results indicate that LTPA is inversely associated with the prevalence of CVD independent of smoking status, and that regular physical activity may protect against CVD for smokers as well as for non-smokers. Physical activity, along with smoking cessation, should be promoted to better prevent and control CVD among smokers.

## Introduction

Cardiovascular disease (CVD) is a major cause of mortality and morbidity worldwide, accounting for 30% of all deaths globally [1,2]. In the U.S., 32.8% (811,940 cases) of all deaths or 1 of every 3 deaths were attributed to CVD in 2008 [3]. Coronary heart disease (CHD) is the largest component of CVD mortality, as 405,309 Americans (~ 1 of every 6 deaths) died of CHD in the same year [3]. It is estimated that each year approximately 785,000 new cases and 470,000 recurrent cases of coronary attack occur in the U.S. [3]. Stroke mortality reportedly was 134,148 in 2008, and nearly 795,000 Americans suffer from a new or recurrent stroke each year [3]. The burden of CVD on healthcare expenditure is substantial, as the total direct and indirect cost associated with CVD in the U.S. was calculated to be $297.7 billion in 2008 [3]. CVD is caused by various biological and behavioral factors [1,2]. In order to effectively prevent and control CVD, it is essential to understand the determinants of CVD as well as the factors that contribute to an increase or decrease in its probability of occurrence.

Cigarette smoking is one of the major risk factors for CVD [4–6]. Smoking was estimated to be responsible for more than 1 in every 10 CVD deaths (1.62 million cases) globally in 2000, accounting for 54% and 25% of CHD and stroke mortality, respectively [7]. As much as 33% of deaths from CVD among American adults aged 35 years or older could be attributed to smoking [8]. Research indicates that smokers are 2–8 times more likely to develop CHD [6,9] and 1.5–4 times more likely to develop stroke than do nonsmokers depending on the presence of other risk factors [10,11]. In particular, studies have shown that smoking is associated with the progression of atherosclerosis [12], lower levels of high-density lipoprotein [13], and decreased exercise tolerance [14]. It was reported that 19.3% (45.3 million) of American adults aged 18 years or older were current smokers in 2010, including 78.2% (35.4 million) of them smoking every day and 21.8% (9.9 million) of them smoking some days [15].

On the other hand, physical activity is known as a protective factor for CVD [16,17]. Evidence shows that regular physical activity can reduce mortality and morbidity associated with CVD [16,17]. Specific benefits of physical activity on preventing CVD include: reducing the risk of atherosclerosis [18], improving plasma lipid profiles (i.e., reductions in low-density lipoprotein cholesterol, very-low-density lipoprotein cholesterol, and triglycerides; increase in high-density lipoprotein cholesterol) [19,20], helping to maintain optimal body weight and decreasing the risk of obesity [21], and preventing and better controlling type 2 diabetes [22]. In addition, engaging in regular physical activity will improve physical fitness [16], as high physical fitness levels are associated with a lower risk of CVD [23,24].

In the state of Nevada, the prevalence of CVD is higher than the prevalence nationwide [25]. In 2010, adult Nevadans who had suffered from heart attack and stroke were 5.0% and 3.1%, respectively, compared to the national averages of 4.2% (heart attack) and 2.7% (stroke) [25]. Furthermore, 21.3% of adult Nevadans were reported to be current smokers (vs. 17.3% nationwide) [25]. Hence, it is essential to prevent and control CVD among people in Nevada, especially for smokers. Although the impacts of cigarette smoking and physical activity on CVD mortality and morbidity are well documented [1,2], it is of interest to understand whether physical activity protects against CVD for smokers in a similar manner as it does for non-smokers. Blair and colleagues [24] reported that physically fit individuals with multiple CVD risk factors, including smoking, elevated blood pressure, and/or elevated cholesterol, had lower mortality than unfit individuals with none of these CVD risk factors. It is reasonably assumed that people with high physical fitness levels tend to engage in regular physical activity. If physical activity significantly reduces the risk of CVD even among people who smoke, they should be encouraged to engage in physical activity as well as to quit smoking for better CVD prevention and control. Meanwhile, since smoking is a well-known risk factor for CVD [4–6], it could be that the association of physical activity to CVD (i.e., benefits of physical activity on reducing the risk of CVD) might not be apparent, if people were smoking. The purpose of this study was to examine how leisure-time physical activity (LTPA) is associated with the prevalence of CVD in relation to smoking status among adult Nevadans.

## Materials and Methods

### Data

Data from the 2010 Nevada Behavioral Risk Factor Surveillance System (BRFSS) were utilized for the current study. Details of the BRFSS are described elsewhere [26]. The BRFSS is a random telephone survey system to collect information on health risk behaviors and self-reported conditions of American adults. It is administered by the Centers for Disease Control and Prevention (CDC), whereas the survey is carried out by state health departments nationwide each year. Non-institutionalized adults aged 18 years or older are randomly selected for interview to collect health related data. In this study, we analyzed the BRFSS data for the state of Nevada collected in 2010. BRFSS data are available in the public domain, allowing researchers to download and analyze data for research studies. The total number of eligible adults identified in the 2010 Nevada BRFSS was 6,114 with the response rate of 50.71% [27].

### Cardiovascular Disease Prevalence

The prevalence of CVD among people was assessed according to each of the following criteria (yes/no): 1) whether they have ever been diagnosed as having a heart attack, 2) whether they have ever been diagnosed as having angina or CHD, and 3) whether they have ever been diagnosed as having a stroke. If an individual answered yes to any one or more of the questions above, they were categorized as having a history of CVD.

### Smoking Status

Respondents were classified based on their smoking status. If an individual had smoked at least 100 cigarettes in his/her entire life and smoked cigarettes every day or some days at the time of the survey, he/she was classified as a current smoker. If an individual had smoked at least 100 cigarettes in his/her entire life but did not smoke at all at the time of the survey, he/she was classified as a former smoker. Lastly, if an individual had never smoked at least 100 cigarettes in his/her entire life, he/she was classified as a non-smoker.

### Leisure-Time Physical Activity

LTPA of people was assessed according to their responses (yes/no) to the following question: “During the past month, other than your regular job, did you participate in any physical activities or exercises such as running, calisthenics, golf, gardening, or walking for exercise?” Based on its response, each individual was categorized as either engaging in LTPA or not engaging in LTPA.

### Data Analysis

Percentages adjusted for sampling weights used in the BRFSS (= weighted percentages) were calculated for smoking status and LTPA, along with selected sociodemographic variables, by CVD prevalence. These weighted percentages were then compared using the Rao-Scott χ^2^ test. A logistic regression analysis was performed to determine an odds ratio (OR) and 95% confidence interval (CI) for smoking status and LTPA (= predictors) by CVD prevalence (= outcome). Gender, age, race/ethnicity, and education as well as body mass index (BMI) and diabetes status were used as covariates in the logistic regression analysis, as these variables have been shown to be associated with the risk of CVD [1,2]. Specifically, BMI was treated as a covariate as a function of LTPA and CVD in the logistic regression model in order to effectively remove its direct impact on CVD, because research shows that physical activity and BMI are independent risk factors for CVD [28,29]. In addition to these analyses, a statistical interaction was examined between LTPA and smoking status on CVD prevalence.

## Results

Data for a total of 3,913 adults were complete and thus analyzed in the current study. Overall, 8.5% of the respondents reported that they had ever been diagnosed with CVD. S1 Table shows sociodemographic characteristics, along with smoking status and LTPA, of the respondents by CVD prevalence. The proportions of those who had ever had CVD were approximately equal between males (49.4%) and females (50.6%). Among people with a history of CVD, higher age was associated with an increased prevalence of CVD (*p* < 0.001). Race/ethnicity was not significantly related to CVD prevalence (*p* = 0.410). Education was found to be a significant variable for CVD prevalence (*p* < 0.001), as 13.6% of the respondents with a history of CVD did not graduate from high school compared with 5.6% of those with no history of CVD. There was a tendency that people with a history of CVD were overweight and obese, though the association between BMI and CVD prevalence was not statistically significant (*p* = 0.096). On the other hand, a significant relationship was observed between diabetes status and CVD prevalence (*p* < 0.001). A percentage of diabetes was higher among people with a history of CVD (25.2%) than among those with no history of CVD (6.9%).

Both smoking status and LTPA were significantly associated with the prevalence of CVD (*p* < 0.001; S1 Table). Close to half of the respondents with a history of CVD were former smokers (48.5%) compared with 23.5% of those with no history of CVD. The proportion of non-smokers among those with a history of CVD was almost as half as that among their counterparts (28.7% vs. 55.4%). People with a history of CVD were significantly less likely to engage in LTPA than those with no history of CVD (53.7% vs. 79.4%).

LTPA by CVD prevalence in relation to smoking status is presented in S2 Table. Overall, 21.4%, 25.8%, and 52.8% of the respondents were current smokers, former smokers, and non-smokers, respectively. Less than 10% of people had ever had CVD among the current (9.0%) and non-smokers (4.6%), while 15.8% of the former smokers had a history of CVD. Regardless of smoking status, people with a history of CVD were significantly less likely to engage in LTPA than those with no history of CVD (*p* < 0.05). The proportions of people engaging in LTPA between those with and without history of CVD were: 43.5% vs. 72.6% among the current smokers (*p* < 0.001), 55.7% vs. 71.4% among the former smokers (*p* = 0.013), and 57.4% vs. 85.4% among the non-smokers (*p* < 0.001).

Odds ratios obtained from the logistic regression analysis are presented in S3 Table. The logistic regression model (logistic model 1 in S3 Table) revealed that after adjusting for gender, age, race/ethnicity, and education as well as BMI and diabetes status, people with a history of CVD were twice more likely to not engage in LTPA than their counterparts independent of smoking status (OR = 2.21, 95% CI = 1.53–3.18). If LTPA was taken into account, the odds of having CVD for the current smokers was not significantly different from the odds for the non-smokers (OR = 1.62, 95% CI = 0.99–2.64). In contrast, the odds of having CVD for the former smokers was still twice as high as the odds for the non-smokers (OR = 2.07, 95% CI = 1.29–3.33).

There was no significant interaction between LTPA and smoking status on CVD prevalence (Wald χ^2^ = 2.124, *p* = 0.346; logistic model 2 in S3 Table). Therefore, the main effects of LTPA and smoking status on CVD prevalence were analyzed as the follow-up analysis (logistic models 3 and 4 in S3 Table). If LTPA was excluded from the logistic regression model, the odds of having CVD for the current and former smokers were 1.87 (95% CI = 1.17–2.97) and 2.25 (95% CI = 1.41–3.59) times higher than the odds for the non-smokers. The main effect of LTPA on CVD prevalence was that people with a history of CVD were overall twice more likely to not engage in LTPA than those with no history of CVD (OR = 2.23, 95% CI = 1.65–3.27).

## Discussion

The results of the data analysis showed that adults living in Nevada who had ever had CVD were less likely to engage in LTPA and more likely to be current and former smokers than those with no history of CVD. These findings were statistically significant even after adjusting for common sociodemographic variables as well as BMI and diabetes status. Moreover, LTPA was inversely associated with the prevalence of CVD independent of smoking status. The analysis also revealed that if LTPA was not accounted for, the odds of having CVD for the current and former smokers were significantly higher than the odds for the non-smokers. However, if the results were accounted for by LTPA, there was no significant difference in the odds of having CVD between the current and non-smokers; whereas, the odds of having CVD for the former smokers was still significantly higher than the odds for the non-smokers. The interaction effect between LTPA and smoking status was not significant.

It was not surprising that both smoking and LTPA were associated with the prevalence of CVD in this study. A number of epidemiological studies have shown that smokers have a higher risk of CVD [5,6,10,11], while a lower CVD risk has been observed among physically active people [16,17]. Furthermore, research indicates that there is a causal relationship between smoking or LTPA and CVD: smoking is a major cause of CVD [4], whereas physical activity protects against developing CVD [16,17]. Evidence also suggests that smoking cessation can significantly reduce the risk of CVD [5], including that of CHD and stroke [11,30], as well as CVD mortality among those who previously had myocardial infarction [31]. Kenfield et al. [32] reported that 5 years of smoking cessation resulted in the reductions in CHD and stroke mortality risks by 61% and 42%, respectively, among middle-aged female cohorts. Regarding the changes in physical activity level and CVD risk, studies have shown that increasing physical activity levels can lower the risk of CVD mortality for healthy individuals as well as for those previously diagnosed with CVD [33–35]. Specifically, increasing physical-activity index to 2,000 kcal or more per week was associated with a 17% decrease in CHD mortality among middle-aged men [33]. According to Greg and colleagues [35], sedentary women who became physically active (physical activity levels equivalent to walking 1 mile per day) showed a reduction in CVD mortality by nearly 40%. Therefore, effective CVD prevention strategies should include promoting both smoking cessation and physical activity, as suggested by the World Health Organization [1,2].

It should be pointed out that there was an inverse relationship between LTPA and CVD prevalence regardless of whether people were current, former, or non-smokers. Interestingly, the odds of having CVD for the current smokers was not significantly different from the odds for the non-smokers after accounting for LTPA. Based on these results, it may be that smoking moderates the relationship between physical activity and CVD despite the nonsignificant interaction between LTPA and smoking status on CVD, as an effect modification (= moderation) can be present without an interaction [36]. Research shows that smoking increases the risk of CVD by 1.5–8 folds in relation to other risk factors [6,9–11]. On the other hand, it has been reported that men and women who regularly engage in leisure-time/moderate-intensity physical activity are about 80–90% and 65–78% less likely to develop CVD, respectively [16]. It could be speculated that LTPA improves the CVD indices worsened by smoking, which will lower the risk of CVD among smokers engaging in physical activity. For example, smoking can increase the risk of atherosclerosis [12], lower the level of high-density lipoprotein [13], and decrease exercise tolerance [14]. In contrast, physical activity can have the opposite effects: decreasing the risk of atherosclerosis [18], increasing the level of high-density lipoprotein [19,20], and improving exercise capacity [16]. Thus, current smokers should be encouraged to participate in regular physical activity as well as to quit smoking for reducing the risk of CVD.

It should be noted that even if LTPA was taken into account, the odds of having CVD for the former smokers was still significantly higher than the odds for the non-smokers. This is not the case for the current smokers whose odds of having CVD compared with the non-smokers became not significantly different after accounting for LTPA. We are uncertain why the association of LTPA to CVD was different between the current and former smokers. One possible reason is that former smokers quit smoking because they had experienced CVD, which would result in a higher CVD prevalence among the former smokers in our study. Another factor could be the sample sizes, as there was relatively a smaller number of current smokers (*N* = 745) compared with former smokers (*N* = 1,324) and non-smokers (*N* = 1,836). As a result, the regression models for the former and non-smokers might be simply better than the model for the current smokers. We also speculate that current smokers may be, in general, more physically active than former smokers. According to recent data [37], the proportion of smokers among adults working in, for example, mining, accommodation and food services, and construction is nearly 30%, which is much higher than the national average of 19.3% in 2010 [15]. It is reasonable to assume that jobs in these industries and occupation groups typically require high physical activity. In addition, blue-collar workers are less likely to succeed in quitting smoking [38]. These factors could lead to a low proportion of blue-collar workers among the former smokers in the current study. In 2010 Nevada BRFSS, physical activity variables other than LTPA were not included. As a result, high physical activity required at work among the current smokers that was not accounted for by LTPA may be one reason why there was a discrepancy in the association of LTPA to CVD between the current and former smokers. Another possibility is that although smoking cessation can reverse the risk of CVD and its benefits can begin immediately [5], Lightwood and Glantz [39] suggest that the decline in CVD risk will level off after about 4 years of quitting smoking, and that relative risks of acute myocardial infarction and stroke will remain above 1.0 thereafter. Consequently, if former smokers did not engage in regular physical activity, their risk of CVD could be still higher than that for non-smokers. Therefore, former smokers should maintain active lifestyles in order to keep their CVD risk low even after they quit smoking.

Limitations associated with the current study should be addressed. First, the data in this study were obtained from self-reports, which is subject to recall bias. For example, the measurement of LTPA relies on a respondent’s cognitive ability to recall his/her behaviors. Second, the 2010 Nevada BRFSS only included the assessment of LTPA. People engage in physical activity other than during leisure-time, such as at work, which, however, was not analyzed in this study. Furthermore, the amounts, modes, and intensities of physical activity done by the respondents were unknown in this study. As a result, we were unable to examine a dose response relation between LTPA and CVD with respect to smoking status. Third, we did not have information on how much current smokers smoked and how long it was since former smokers had quit smoking in this study. Lastly, this is an observational, cross-sectional study; thus, there could be other confounding factors and moderator/mediator variables influencing smoking status, LTPA, and/or CVD that were not taken into account in the study. This makes challenging to isolate the effect of a single variable and to interpret the results of the data analysis, especially for the interaction effect. We included BMI and diabetes status, along with sociodemographic variables, as covariates in the data analysis. Meanwhile, other variables, such as hypertension and high cholesterol that are known CVD risk factors [1,2], were not available in the 2010 Nevada BRFSS, and therefore not controlled for in the study. Furthermore, although physical activity is known to protect CVD [16,17], we were unable to identify higher levels of evidence indicating that physical activity causes a reduction of CVD among smokers. There is also a possibility of reverse causation between LTPA and CVD when smoking status is taken into account.

Smoking prevalence has declined by more than 20% over the past four decades [40,41], which helps to prevent and control CVD [1,2]. Yet, physical inactivity/sedentary behavior is still prevalent in the general population, as more than 1 out of 3 adults reported that they engaged in no LTPA in 2008 [42]. Since our study shows that physical activity could significantly reduce the risk of CVD even if people smoke, public health professionals should strive to promote physical activity whilst continuing to raise awareness of the health risks caused by smoking. This is imperative especially for the state of Nevada because of its higher smoking rate and CVD prevalence [25]. We hope that the current trend of physical inactivity will be reversed, similar to the decreased smoking rates we have seen over the years,

## Conclusions

The present study has highlighted the importance of regular physical activity among current and former smokers as well as non-smokers for reducing the risk of CVD. The results of the study indicate that LTPA is inversely associated with the prevalence of CVD independent of smoking status, and that regular physical activity may protect against CVD for smokers as well as for non-smokers. Hence, people regardless of smoking status should engage in regular physical activity in order to reduce the risk of CVD. This is especially important for smokers, as their CVD risk is already higher than that for non-smokers. From the public health perspective, physical activity promotion in addition to smoking cessation should be an essential part of CVD prevention and control. In clinical practice, the findings of this study encourage healthcare professionals to recommend regular physical activity, along with smoking secession, in order to reduce CVD mortality for smokers. Moreover, our findings may change how clinicians and practitioners advise their patients in terms of reducing the risk of CVD by putting more emphasis on being active rather than simply advising to quit smoking.

## Supporting Information

S1 Dataset SAS data file for the 2010 Nevada Behavioral Risk Factor Surveillance System. (SAS7BDAT)

S1 Table Sociodemographic characteristics, smoking status, and leisure-time physical activity by cardiovascular disease prevalence. Notes: values given as % (SE); CVD = cardiovascular disease; BMI = body mass index; LTPA = leisure-time physical activity. ^*^Rao-Scott χ^2^ test.
(DOCX)

S2 Table Leisure-time physical activity by cardiovascular disease prevalence in relation to smoking status. Notes: values given as % (SE); CVD = cardiovascular disease; LTPA = leisure-time physical activity. ^*^Rao-Scott χ^2^ test.
(DOCX)

S3 Table Odds ratios and interaction effect based on logistic regression models for leisure-time physical activity and smoking status by cardiovascular disease prevalence. Notes: CVD = cardiovascular disease; LTPA = leisure-time physical activity; OR = odds ratio; CI = confidence interval. ^*^Reference category. ^**^Since there was no significant interaction between LTPA and smoking status, only the main effects of LTPA and smoking status on CVD prevalence were analyzed (logistic regression models 3 and 4).
(DOCX)
