# Supplementary material for: Association of Leisure-Time Physical Activity to Cardiovascular Disease Prevalence in Relation to Smoking among Adult Nevadans
Source: PLoS One. 2015 May 26;10(5):e0128424. doi: 10.1371/journal.pone.0128424 (PMC4444111; doi:10.1371/journal.pone.0128424)
Supplement: S1 Table — Notes: values given as % (SE); CVD = cardiovascular disease; BMI = body mass index; LTPA = leisure-time physical activity. *Rao-Scott χ2 test. (DOCX) [file pone.0128424.s002.docx]

**Table S1: Sociodemographic characteristics, smoking status, and leisure-time physical activity by cardiovascular disease prevalence.**

|  | History of CVD (*N* = 3,913) | | |
| --- | --- | --- | --- |
|  | Yes | No |  |
| Characteristic | 8.5 (0.6) | 91.5 (0.6) | *p*-value^*^ |
| Gender |  |  | 0.776 |
| Male | 49.4 (3.6) | 50.5 (1.5) |  |
| Female | 50.6 (3.6) | 49.5 (1.5) |  |
| Age |  |  | < 0.001 |
| 18-29 years | 4.5 (3.0) | 22.0 (1.6) |  |
| 30-39 years | 2.4 (1.3) | 20.9 (1.4) |  |
| 40-49 years | 10.1 (2.3) | 21.6 (1.2) |  |
| 50-59 years | 22.4 (3.1) | 14.9 (0.9) |  |
| 60-69 years | 29.2 (3.2) | 11.9 (0.7) |  |
| ≥ 70 years | 31.4 (3.0) | 8.7 (0.5) |  |
| Race/ethnicity |  |  | 0.410 |
| White | 70.2 (3.8) | 67.4 (1.6) |  |
| Black | 5.7 (1.4) | 5.8 (0.8) |  |
| Hispanic | 11.0 (3.3) | 16.2 (1.4) |  |
| Other | 13.1 (2.7) | 10.6 (1.1) |  |
| Education |  |  | < 0.001 |
| Did not graduate from high school | 13.6 (2.5) | 5.6 (0.6) |  |
| Graduated from high school | 26.6 (3.1) | 32.0 (1.5) |  |
| Attended college/technical school | 37.6 (3.7) | 32.0 (1.4) |  |
| Graduated from college/technical school | 22.2 (2.8) | 30.4 (1.4) |  |
| BMI (kg/m^2^) |  |  | 0.096 |
| Not overweight/obese | 32.6 (3.4) | 40.5 (1.5) |  |
| Overweight | 39.4 (3.8) | 36.8 (1.5) |  |
| Obese | 28.0 (3.1) | 22.7 (1.3) |  |
| Diabetes |  |  | < 0.001 |
| Yes | 25.2 (3.1) | 6.9 (0.7) |  |
| No | 74.8 (3.1) | 93.1 (0.7) |  |
| Smoking status |  |  | < 0.001 |
| Current smokers | 22.8 (3.0) | 21.1 (1.2) |  |
| Former smokers | 48.5 (3.7) | 23.5 (1.2) |  |
| Non-smokers | 28.7 (3.2) | 55.4 (1.5) |  |
| LTPA |  |  | < 0.001 |
| Yes | 53.7 (3.7) | 79.4 (1.2) |  |
| No | 46.3 (3.7) | 20.6 (1.2) |  |

Notes: values given as % (SE); CVD = cardiovascular disease; BMI = body mass index; LTPA = leisure-time physical activity.

^*^Rao-Scott χ^2^ test.
