# Supplementary material for: Association of Leisure-Time Physical Activity to Cardiovascular Disease Prevalence in Relation to Smoking among Adult Nevadans
Source: PLoS One. 2015 May 26;10(5):e0128424. doi: 10.1371/journal.pone.0128424 (PMC4444111; doi:10.1371/journal.pone.0128424)
Supplement: S2 Table — Notes: values given as % (SE); CVD = cardiovascular disease; LTPA = leisure-time physical activity. *Rao-Scott χ2 test. (DOCX) [file pone.0128424.s003.docx]

**Table S2: Leisure-time physical activity by cardiovascular disease prevalence in relation to smoking status.**

|  | Current smokers (*N* = 745) | | |  | Former smokers (*N* = 1,324) | | |  | Non-smokers (*N* = 1,836) | | |
| --- | --- | --- | --- | --- | --- | --- | --- | --- | --- | --- | --- |
|  | 21.4 (1.2) | | |  | 25.8 (1.2) | | |  | 52.8 (1.4) | | |
|  | History of CVD | | |  | History of CVD | | |  | History of CVD | | |
|  | Yes | No |  |  | Yes | No |  |  | Yes | No |  |
| LTPA | 9.0 (1.3) | 91.0 (1.3) | *p*-value^*^ |  | 15.8 (1.7) | 84.2 (1.7) | *p*-value^*^ |  | 4.6 (0.6) | 95.4 (0.6) | *p*-value^*^ |
| Yes | 43.5 (7.3) | 72.6 (2.7) | < 0.001 |  | 55.7 (5.4) | 71.4 (3.2) | 0.013 |  | 57.4 (6.7) | 85.4 (1.4) | < 0.001 |
| No | 56.5 (7.3) | 27.4 (2.7) |  |  | 44.3 (5.4) | 28.6 (3.2) |  |  | 42.6 (6.7) | 14.6 (1.4) |  |

Notes: values given as % (SE); CVD = cardiovascular disease; LTPA = leisure-time physical activity.

^*^Rao-Scott χ^2^ test.
