# Supplementary material for: Association of Leisure-Time Physical Activity to Cardiovascular Disease Prevalence in Relation to Smoking among Adult Nevadans
Source: PLoS One. 2015 May 26;10(5):e0128424. doi: 10.1371/journal.pone.0128424 (PMC4444111; doi:10.1371/journal.pone.0128424)
Supplement: S3 Table — Notes: CVD = cardiovascular disease; LTPA = leisure-time physical activity; OR = odds ratio; CI = confidence interval. *Reference category. **Since there was no significant interaction between LTPA and smoking status, only the main effects of LTPA and smoking status on CVD prevalence were analyzed (logistic regression models 3 and 4). (DOCX) [file pone.0128424.s004.docx]

**Table S3: Odds ratios and interaction effect based on logistic regression models for leisure-time physical activity and smoking status by cardiovascular disease prevalence.**

|  | History of CVD (= yes) | | | | | | |
| --- | --- | --- | --- | --- | --- | --- | --- |
|  | Logistic model 1 |  | Logistic model 2 |  | Logistic model 3 |  | Logistic model 4 |
| Predictor | OR (95% CI) |  | Interaction effect |  | OR (95% CI) |  | OR (95% CI) |
| LTPA |  |  | LTPA × smoking status  (Wald χ^2^ = 2.124, *p* = 0.346)^**^ |  |  |  |  |
| Yes^*^ vs. No | 2.21 (1.53–3.18) |  |  |  | Excluded |  | 2.32 (1.65–3.27) |
| Smoking status |  |  |  |  |  |  |  |
| Non-smoker^*^ vs. current smoker | 1.62 (0.99–2.64) |  |  |  | 1.87 (1.17–2.97) |  | Excluded |
| Non-smoker^*^ vs. former smoker | 2.07 (1.29–3.33) |  |  |  | 2.25 (1.41–3.59) |  |  |

Notes: CVD = cardiovascular disease; LTPA = leisure-time physical activity; OR = odds ratio; CI = confidence interval.

^*^Reference category.

^**^Since there was no significant interaction between LTPA and smoking status, only the main effects of LTPA and smoking status on CVD prevalence were analyzed (logistic regression models 3 and 4).
